# Measurement of CSF core Alzheimer disease biomarkers for routine clinical diagnosis: do fresh vs frozen samples differ?

**DOI:** 10.1186/s13195-020-00689-0

**Published:** 2020-09-29

**Authors:** Giovanni Bellomo, Samuela Cataldi, Silvia Paciotti, Federico Paolini Paoletti, Davide Chiasserini, Lucilla Parnetti

**Affiliations:** 1 Laboratory of Clinical Neurochemistry, Section of Neurology, University of Perugia, Piazzale Lucio Severi 1/8, 06132 Perugia, PG Italy; 2 Department of Experimental Medicine, Section of Physiology and Biochemistry, University of Perugia, Piazza Lucio Severi 1/8, 06132 Perugia, PG Italy; 3 Section of Neurology, University of Perugia, Piazzale Lucio Severi 1/8, 06132 Perugia, PG Italy

**Keywords:** Biomarkers, Alzheimer’s disease, Cerebrospinal fluid, Automated platforms, Pre-analytical variables

## Abstract

**Background:**

Cerebrospinal fluid (CSF) amyloid-beta (Aβ) 42/40 ratio, threonine-181-phosphorylated-tau (p-tau), and total-tau (t-tau) represent core biomarkers of Alzheimer disease (AD). The recent availability of automated platforms has represented a significant achievement for reducing the pre-analytical variability of these determinations in clinical setting. With respect to classical manual ELISAs, these platforms give us also the possibility to measure any single sample and to get the result within approximately 30 min. So far, reference values have been calculated from measurements obtained in frozen samples. In this work, we wanted to check if the values obtained in fresh CSF samples differ from those obtained in frozen samples, since this issue is mandatory in routine diagnostic work.

**Methods:**

Fifty-eight consecutive CSF samples have been analyzed immediately after lumbar puncture and after 1-month deep freezing (− 80 °C). As an automated platform, we used Lumipulse G600-II (Fujirebio Inc.). Both the fresh and the frozen aliquots were analyzed in their storage tubes.

**Results:**

In fresh samples, a mean increase of Aβ40 (6%), Aβ42 (2%), p-tau (2%), and t-tau (4%) was observed as compared to frozen samples, whereas a slight decrease was observed for Aβ42/Aβ40 ratio (4%), due to the higher deviation of Aβ40 in fresh samples compared to Aβ42. These differences are significant for Aβ40, Aβ42/Aβ40 ratio, p-tau, and t-tau. Nevertheless, the Aβ42/Aβ40 ratio showed a lower variability (smaller standard deviation of relative differences) with respect to Aβ42. With respect to the AD profile according to the A/T/(N) criteria for AD diagnosis, no significant changes in classification were observed when comparing results obtained in fresh vs frozen samples.

**Conclusions:**

Small but significant differences have been found for Aβ40, Aβ42/Aβ40 ratio, p-tau, and t-tau in fresh vs frozen samples. Importantly, these differences did not imply a modification in the A/T/(N) classification system. In order to know if different cutoffs for fresh and frozen samples are required, larger, multi-center investigations are needed.

## Background

Cerebrospinal fluid (CSF) amyloid-beta 42 (Aβ42), Aβ40, Aβ42/Aβ40 ratio, threonine-181-phosphorylated-tau (p-tau), and total-tau (t-tau) are reliable biomarkers for amyloidosis (A), tauopathy (T), and neurodegeneration (N). These biomarkers are able to identify Alzheimer’s disease (AD) independent of the clinical stage, thus, including the preclinical stages [1, 2] and mild cognitive impairment (MCI) [3]. Also, they are reliable predictors of progression to dementia [4] up to or more than 10 years before [5]. The application of CSF biomarkers in clinical practice has been strongly encouraged by the NIA-AA (National Institute on Aging and the Alzheimer’s Association) [6], in order to promote the shift of the definition of AD from a syndromic to a biological construct.

The classical way to measure AD biomarkers is through manual Enzyme-Linked Immunosorbent Assay (ELISA) [7], which suffers from user- and laboratory-dependent procedures that may cause reproducibility issues [8, 9]. Manual ELISA is carried out in 96-well plates, which often requires to wait for an adequate number of samples available, in order to avoid waste of materials. Moreover, intra-assay variability also affects ELISA, due to the time required to fulfill the plate. The standardization of pre-analytical and analytical procedures (i.e., freeze/thaw cycles [10], CSF storage volumes [10], pipette-tips and tube materials [11, 12], storage temperature [13], and variability due to the operator [14]) represents a crucial factor in biomarker assays [15]. In this context, automated [16–20] and semi-automated [21] platforms may play a major role in minimizing inter-laboratory differences in biomarker assays. Fully automated chemiluminescent platforms [20] showed high sensitivity and specificity for early diagnosis of AD, with an optimal concordance with manual ELISA assays [18, 20] and amyloid-targeted positron emission tomography (PET) [19, 22].

Interestingly, these platforms offer the possibility to easily and quickly analyze any single, freshly collected CSF sample. This opportunity has two major advantages: (1) it may allow to rule out in real time the diagnosis of subacute encephalitis, namely Creutzfeldt-Jacob disease, and (2) in expert centers collecting every day CSF samples for diagnostic purposes, it might be useful/appropriate to get the result in the same day, as also possible in other medical services/specialties.

So far, reference values are represented by measurements obtained in samples aliquoted and frozen. The perspective to analyze fresh CSF samples made us wonder if biomarker measurements can differ between fresh and frozen samples. If yes, then new cutoffs should be defined for fresh samples. Accordingly, in this work, we evaluated the values of Aβ42, Aβ40, Aβ42/Aβ40, t-tau, and p-tau, obtained in fresh and after 1-month freezing CSF samples.

## Methods

### Lumbar puncture and general CSF handling

CSF samples were obtained from a consecutive series of out-patients referring to Center of Memory Disturbances of the University of Perugia for routine diagnostic work-up and collected according to international guidelines [23–25]. All patients gave their informed written consent. All the procedures were performed following the Helsinki Declaration. Lumbar punctures were performed from 8:00 to 10:00, after an overnight fasting. CSF (~ 12 mL) was immediately collected in sterile polypropylene tubes (Sarstedt® tubes, codes: 62.610.210) and gently mixed to avoid possible gradient effects. All samples were centrifuged at 2000×*g* for 10 min, at room temperature, and then aliquoted in 0.5 mL aliquots in sterile polypropylene tubes (Sarstedt® tubes, codes: 72.730.007).

### Biomarker assay on fresh and frozen CSF samples

**Fig. 1:**
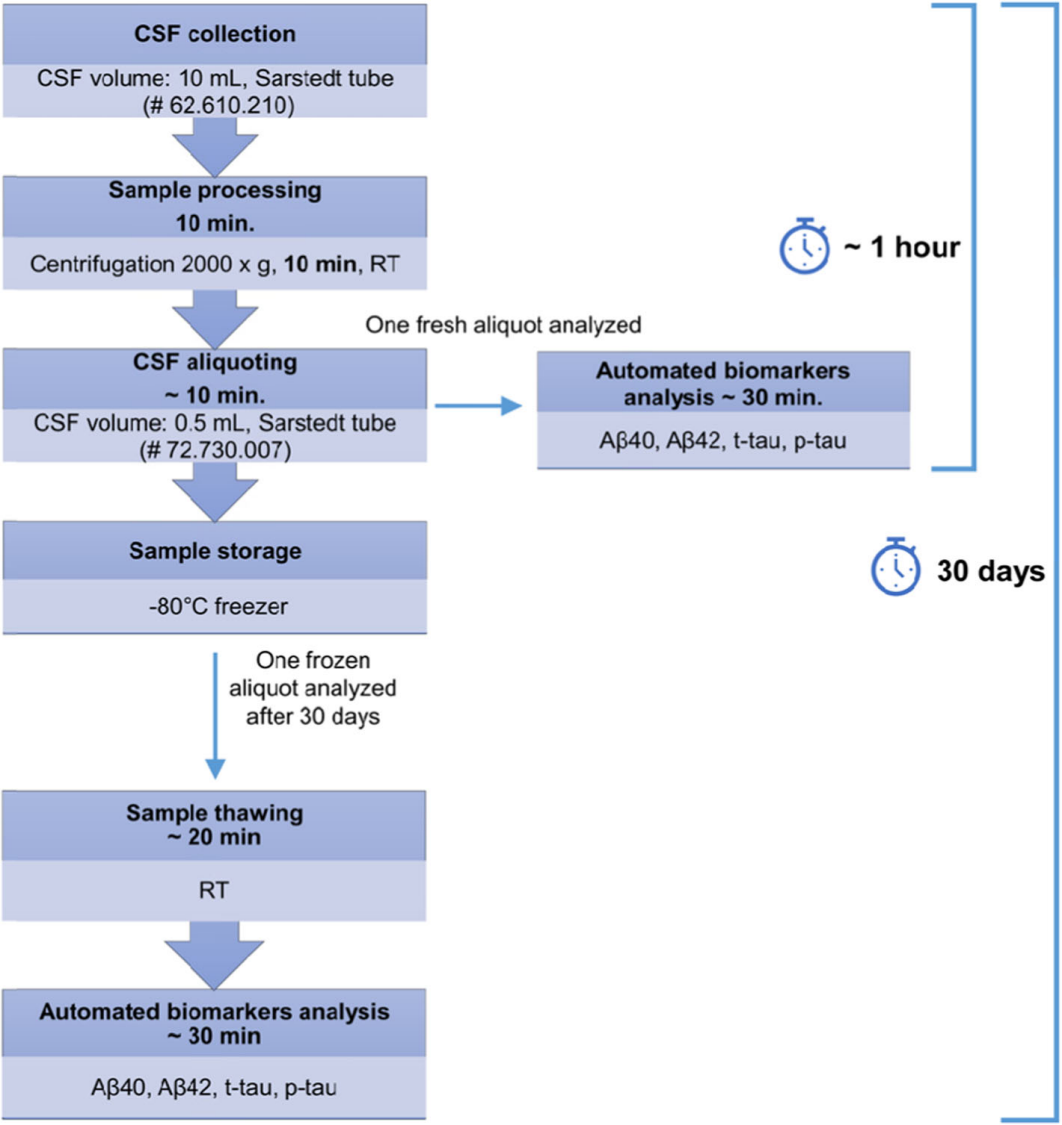
Scheme of the experimental workflow followed to test the impact of fresh vs frozen CSF using automated platform

Fifty-eight CSF samples (28 males and 30 females, mean age 70.3 years, SD 7.8 years) were consecutively collected from patients and processed as described above. Patient data (sex, age, CSF collection date, diagnosis, and biomarker levels) are included in the supporting information in Table S3. For each sample, one aliquot was immediately analyzed in its 0.5 mL Sarstedt® tube on the Lumipulse G600-II (Fujirebio inc.) for Aβ40, Aβ42, t-tau, and p-tau, while the others were frozen at − 80 °C. We analyzed a second aliquot after 30 days of freezing at − 80 °C, using the same methodology. The experimental workflow of this study is summarized in Fig. 1. Apart from the quality controls (QC) samples included in the kits, an internal quality control (QC), consisting of a pool of CSF samples, has been analyzed during each run. The type of the QC sample used, mean biomarker values, standard deviations, and coefficients of variation (CV) are reported in Table S4 in the supporting information.

### Statistical analysis

To evaluate the impact of freezing, biomarkers values measured on fresh samples were compared to the ones obtained from another aliquot of the same sample after 1-month storage at − 80 °C. Due to the non-normality of biomarker data, Passing Bablok regressions [26] were performed instead of parametric least squares regressions. Confidence intervals (CI) for the fitted parameters were calculated with the bootstrap method [27]. Correlations between biomarkers measured in fresh and frozen samples were estimated in terms of Spearman’s correlation coefficients (*ρ*). Bland-Altman plots [28] with trends and CI were also generated with the R-package *blandr* [29]. To assess the significance of the observed differences, we computed the relative difference *ΔB* between freshly acquired (*B*_fresh_) and post-freezing (*B*_frozen_) values of y Aβ40, Aβ42, Aβ40/Aβ42 ratio, p-tau, and t-tau, from two aliquots originating from the same CSF sample.

$$ \varDelta B=\frac{B_{\mathrm{fresh}}-{B}_{\mathrm{frozen}}}{B_{\mathrm{fresh}}} $$

Kolmogorov-Smirnov normality test could not reject the normality of the *ΔB* values for each tested biomarker, thus one-sample Student’s *t* test was applied to assess the significance of the relative differences in biomarkers between fresh and frozen CSF samples. A *p* value below 0.05 was considered significant to reject the null hypothesis. Cutoff values for fresh samples were calculated using both regression analysis [30] and by maximizing Youden’s index with the p-ROC package in R [31]. For the regression transfer method, cutoffs CI were calculated from the ones obtained for the coefficients of the Passing Bablok regression, while for the Youden’s index maximization, CI were calculated using 2000 bootstrap replicates.

### Biomarker classification

In order to appreciate the variability of fresh vs frozen CSF aliquots, we compared the NIA-AA A/T/(N) classification criteria using standard cutoffs. These standards, developed in our laboratory, are also reported in the biomarker assay cartridge datasheets of the Lumipulse G600-II. Biomarker values were classified as positive (+) or negative (−) by using the cutoff values of 0.069 for Aβ42/Aβ40 ratio, 56.5 pg/mL for p-tau, and 404 pg/mL for t-tau. Samples positive for both Aβ42/Aβ40 ratio and p-tau (A+/T+) have been classified as AD [6] (raw classification). A more robust classification was also applied by classifying AD or non-AD CSF profiles considering a tolerance of ± 10% on the cutoff values of Aβ42/Aβ40 ratio and p-tau. This tolerance is usually considered in routine diagnostics in order to overcome inter-assay variability.

## Results

**Fig. 2:**
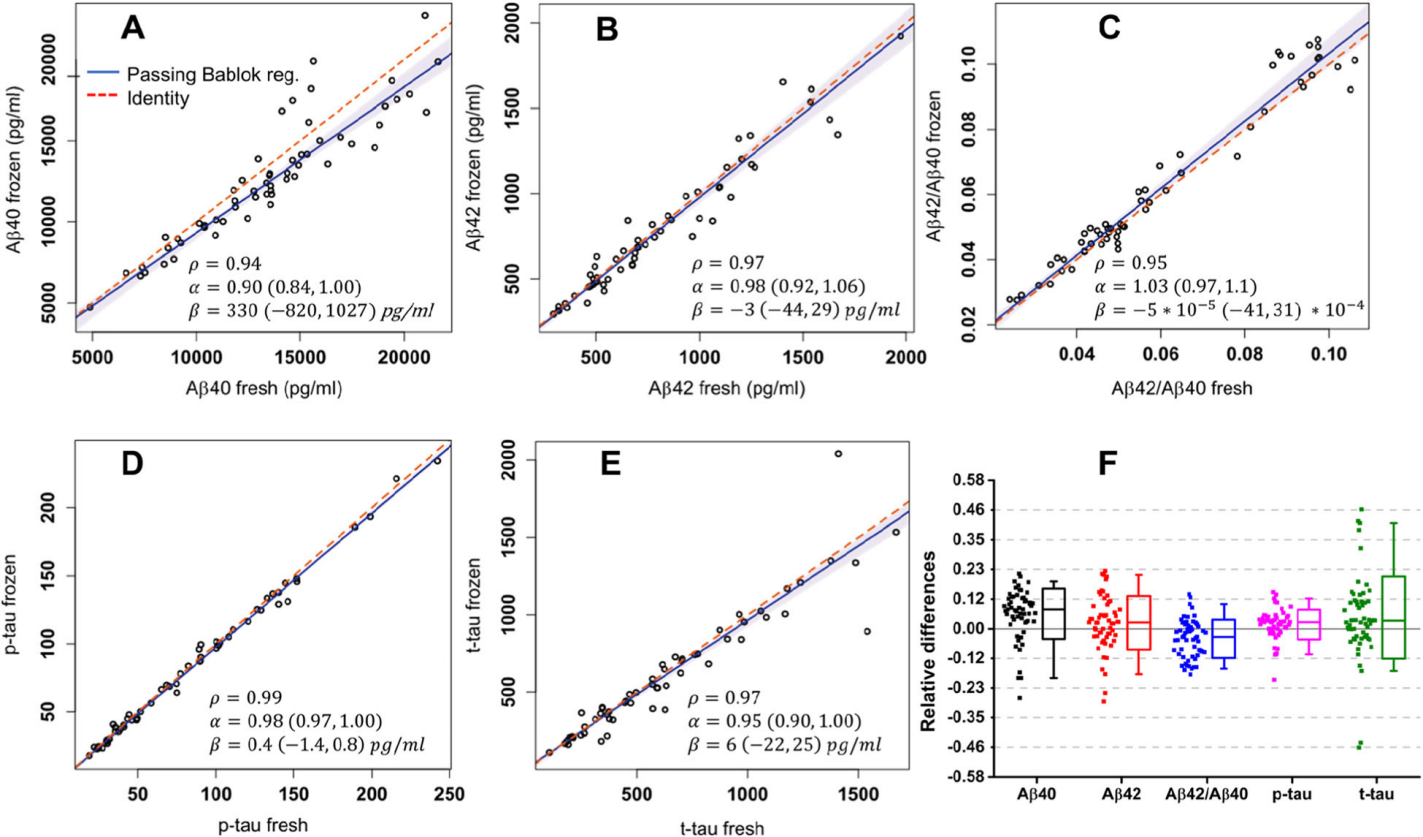
**a**–**e** Passing Bablok regression analyses with 95% CI of core biomarkers measured in fresh CSF samples and after 1 month of deep-freezing. Correlations have been calculated in terms of Spearman’s correlation coefficients (ρ). Fitted slopes (α) and intercepts (β) with their 95% CI are also shown. **f** Mean relative differences of Aβ40, Aβ42, Aβ42/Aβ40, t-tau, and p-tau between fresh and frozen samples are plotted together. Boxes representing data distributions are centered on the mean values, with the internal horizontal line representing the median. Boxes heights are equal to the standard deviations of the relative differences while whiskers represent the 5–95% data range

The levels of Aβ40, Aβ42, p-tau, and t-tau were measured with a fully automated chemiluminescent platform (Lumipulse G600-II, Fujirebio Inc.) on two aliquots of the same CSF sample, one analyzed immediately after the sampling (“fresh”) and the other after 30 days of storage at − 80 °C. To investigate the relation between fresh and frozen biomarker levels, Passing Bablok regressions were performed, the results of this analysis are plotted in Fig. 2a–e. To assess the significance of the deviations from identity, the relative differences on Aβ40, Aβ42, Aβ42/Aβ40, t-tau, and p-tau were also calculated, and the results are represented in Fig. 2f.

As shown in the panels a–e, fitted intercepts are consistently equal to zero within their CI for all the measured quantities. The largest deviation from identity was obtained for Aβ40. Bland Altman plots (Fig. S1) showed higher concentration values for Aβ40, Aβ42, p-tau, and t-tau in fresh samples and a trend of larger differences in magnitude for higher values of biomarkers. However, about 95% of the samples fell within 2 SD from the mean fresh vs frozen difference, confirming the high correlation obtained with Passing-Bablok analysis. As shown in Fig. 2f and Table 1, mean relative differences greater than 0.06 have not been observed for any of the tested biomarkers. Even if small, the differences on Aβ40, Aβ42/Aβ40 ratio, t-tau, and p-tau were significant according to Student’s *t* test (see Table 1). Although the relative differences on Aβ42/Aβ40 ratio were significant, this biomarker showed less variability in terms of standard deviation (SD) of relative differences with respect to Aβ42 and Aβ40 alone (0.075 vs 0.11). The highest SD of relative differences was obtained for t-tau (0.16) while the smallest for p-tau (0.06); the latter showed also the smallest mean relative difference (0.017) and the highest correlation between measurements (*ρ* = 0.99). Despite of the presence/absence of the freezing step, for each analyte, the measured SD of relative differences between fresh and frozen samples was of the same order of magnitude of the inter-assay variability (CV) of the QC sample (Table S4).

**Table 1:** Mean relative differences between fresh and frozen samples with their 95%CI, standard deviations of relative differences (SD), and *p* values obtained by applying one-sample Student’s *t* test

Biomarker	Mean relative difference (95%CI)	SD	*p* values *t* test
Aβ40	0.059 (0.033, 0.084)	0.10	< 0.001
Aβ42	0.023 (− 0.004, 0.051)	0.11	0.10
Aβ42/Aβ40	− 0.039 (− 0.059, − 0.020)	0.075	< 0.001
t-tau	0.044 (0.001, 0.086)	0.16	0.04
p-tau	0.017 (0.0015, 0.032)	0.059	0.03

For each aliquot, Aβ42/Aβ40, p-tau, and t-tau have been classified according to the A/T/(N) criteria [6] by using standard cutoffs and tolerances (reported in the “Methods” section). The results of this classification are shown in Table 2.

**Table 2:** Classification of core AD biomarkers and in fresh and frozen samples by using the A/T/(N) criteria. Number of changes in classification between biomarkers measured in fresh and stored at − 80 °C CSF samples are also reported. Samples with both pathologic Aβ42/Aβ40 ratio and pathologic p-tau (A+/T+) have been classified positive for AD. For “robust” AD diagnosis and AD exclusion (non-AD) a ± 10% tolerance was applied on the cutoffs of Aβ42/Aβ40 ratio and p-tau

	+ (%)	− (%)	No. of changes (%)
Aβ42/Aβ40 frozen	39 (67%)	19 (33%)	1 (2%)
Aβ42/Aβ40 fresh	40 (69%)	18 (31%)	
p-tau frozen	34 (59%)	24 (41%)	0 (0%)
p-tau fresh	34 (59%)	24 (41%)	
t-tau frozen	34 (59%)	24 (41%)	2 (3%)
t-tau fresh	36 (62%)	22 (38%)	
AD frozen	34 (59%)	24 (41%)	0 (0%)
AD fresh	34 (59%)	24 (41%)	
Robust AD frozen	32 (55%)	26 (45%)	1 (2%)
Robust AD fresh	33 (57%)	25 (43%)	
Robust non-AD frozen	24 (41%)	34 (59%)	1 (2%)
Robust non-AD fresh	23 (40%)	35 (60%)	

Considering the classification (Table 2), in some cases, Aβ42/Aβ40 ratio and t-tau changed their classification between fresh and frozen samples. However, when looking at AD diagnosis, which mainly relies on the values of Aβ42/Aβ40 ratio and p-tau (A/T), none of the samples changed classification directly from + to − or the other way around considering the raw cutoffs. Only one sample changed from + to − by considering a + 10% tolerance on Aβ42/Aβ40 ratio and p-tau cutoffs (robust AD diagnosis) and another one when considering a − 10% for excluding AD.

To further investigate the impact of these differences in diagnostics, we recalculated the cutoffs for Aβ42/Aβ40 ratio, p-tau, and t-tau for fresh samples. One way to do that is to transfer frozen-defined cutoffs by applying a linear transformation based on the parameters estimated with the Passing Bablok regression (cutoff fresh R in Table 3). Another way can be to consider the raw classification of frozen samples as a reference and maximize Youden’s index for fresh samples (cutoff fresh Y in Table 3). The results of both procedures are shown in Table 3.

**Table 3:** Recalculated cutoffs for freshly measured CSF core AD biomarkers with their 95% CI. Cutoff fresh R: cutoff transferred by applying Passing Bablok regression. Cutoff fresh Y: cutoff calculated maximizing the Youden’s index for fresh samples, by taking as reference the classification performed on frozen ones

Biomarker	Cutoff frozen	Cutoff fresh R (95%CI)	Cutoff fresh Y (95%CI)
Aβ42/Aβ40	0.069	0.067 (0.060, 0.075)	0.062 (0.061, 0.075)
p-tau	56.5	57.2 (55.7, 59.7)	55.1 (52.3, 60.9)
t-tau	404	419 (379, 473)	419 (406, 581)

As it is shown in Table 3, only for t-tau the recalculated cutoff from direct diagnostic information (Youden) does not include the cutoff defined in frozen samples within its 95% CI.

## Discussion

Compared to traditional manual ELISAs, automated chemiluminescent platforms allow the measurement of biomarkers on CSF samples with minimal handling and quick results, including the possibility to analyze any single freshly collected CSF sample. As a consequence, results in real time can be obtained in case of clinical suspect of subacute encephalitis, or as a proof of high-level diagnostic performance in expert centers dedicated to early AD diagnosis.

For this purpose, we evaluated if core AD biomarkers, namely Aβ40, Aβ42, Aβ42/Aβ40, p-tau, and t-tau, differ from fresh to frozen CSF samples and if new cutoffs should be defined for freshly measured biomarkers. The effect of freezing on biomarker measurements was previously tested by Sjögren et al. [32]. No significant differences were measured in Aβ42 and t-tau between fresh and one-time thawed samples by ELISAs. However, only 8 samples were tested and thus the significance of small variations could have been hindered by the limited sample size. Similar considerations can be done also for the more recent works of Le Bastard et al. [9] and Janelidze et al. [33], in which, together with other preanalytical factors, the impact of freezing was tested for Aβ42, p-tau, and t-tau and for Aβ40, Aβ42, and Aβ42/Aβ40 ratio, respectively, on 10 samples by ELISAs. In our experiments, by using aliquots originating from 58 distinct patients, small but significant systematic differences were observed for Aβ40, Aβ42/Aβ40, p-tau, and t-tau between fresh and frozen samples, while a non-significant decreased value of Aβ42 was also measured. The slight decrease in concentration of the tested proteins on frozen samples may have been produced by an unspecific loss caused by tube adsorption [10, 34] or by a slight degradation during the freezing/thaw step caused by residual proteases. Among the tested proteins, Aβ40 showed the largest decrease (6%). Aβ40 is a naturally unfolded peptide, which makes it very accessible to the solvent, and thus also very small amounts of proteases may produce an appreciable degradation [35]. We questioned if a release of proteases may have been produced by the lysis of residual cells in CSF but, as can be evinced from Table S1, no significant differences in variability were observed for samples containing different number of cells with the exception of Aβ42 that, however, did not show significant variations upon freezing. The decrease of Aβ40 altered also the value of AB42/Aβ40 ratio. In routine clinical practice, Aβ42/Aβ40 ratio is usually preferable to Aβ42 alone [36, 37] and, although it showed a greater mean relative difference between fresh and frozen samples with respect to Aβ42, it also showed a lower SD of relative differences between values measured in fresh and frozen CSF samples. With respect to the A/T/(N) classification, whereas for some CSF samples with borderline profiles Aβ42/Aβ40 ratio and t-tau changed classification, while considering the diagnostic criteria for AD, none of the samples changed diagnosis in a raw dichotomous classification. By taking into account a tolerance of ± 10% on cutoffs for confirming or excluding AD, only two samples (2%), one for the confirmation and one for the exclusion, changed in classification. We successively transferred the cutoff values to fresh samples by applying two of the three methods described in the work of Barrado et al. [30]. Due to the non-normality of biomarker values, we could not apply the *Bayesian two-stage cutoff transfer* method. For the *Linear-regression-based transfer* of the cutoff value, we bypassed the normality assumption by using the coefficients obtained through the Passing Bablok regression, while for the *direct cutoff estimation from diagnostic information*, we determined the cutoffs that maximized Youden’s index in fresh samples by using the classification of frozen samples as reference. Both methods have pros and cons; the regression-based method utilizes all the data information but it may produce biased results when unequal populations of high and low biomarker values are present. Conversely, the direct estimation from diagnostic information is more robust but it may suffer from the non-optimal diagnostic representativeness of our samples. The two methods produced similar results and generally included frozen-defined cutoffs within 95% CI. The only exception is the cutoff obtained for t-tau from direct diagnostic information (Youden) that did not include the frozen-defined cutoff within its CI. However, considering the variability of biomarkers among measurements, the number of tested samples (*N* = 58) may have been insufficient to prove or disprove a real discrepancy between frozen-defined and fresh-defined cutoffs.

## Conclusions

The wide availability of automated platforms represents an important achievement for reducing the pre-analytical variability of core AD biomarker assays in routine clinical setting [20]. With respect to classical manual ELISAs, automated platforms give us also the possibility to measure single samples and to obtain the results within approximately 30 min. We wanted to check if the values obtained in fresh CSF differ from those obtained in frozen samples. Although some differences were found for Aβ40, Aβ42/Aβ40 ratio, p-tau, and t-tau in fresh vs frozen samples, cutoff values for AD diagnosis recomputed on fresh samples did not significantly differ from those obtained in frozen samples. These results are encouraging. However, in order to definitely rule out the need of specific cutoffs for fresh CSF samples, larger, multi-center investigations are recommended.

## Supplementary information

**Additional file 1: Table S1.** Mean absolute relative differences (MARD) in samples with no cells and 1-2 cells. The measured MARD are higher for samples with 1-2 cells but this difference is significant only for Aβ42. **Table S2.** Linear regression analysis performed on Mean absolute relative differences (MARD) vs CSF total proteins. No significant correlations were found. **Figure S1.** Bland-Altman Plot of differences between fresh and frozen CSF aliquots of the same samples vs. the mean of the two measurements (data from Table 1). Shaded areas present 95% confidence interval limits for mean and agreement limits. **Table S3.** patient sex, patient age, sample internal biobank code, lumbar puncture (LP) date and raw biomarker measurements in fresh and frozen samples. Two aliquots relative to the same CSF sample were measured with Lumipulse G-600 II, one by fresh and the other one after 30 days of storage at -80°C. AD: Alzheimer’s disease; MCI: mild cognitive impairment; MCI-AD: MCI due to AD; p-AD: preclinical AD; CBS: corticobasal syndrome; V-DEM: vascular dementia; PD: Parkinson’s disease; PD-MCI: PD with MCI; PDD: PD with dementia; DLB: dementia with Lewy bodies; FTD: frontotemporal dementia; SMC: subjective memory complains; PSY: psychiatric disease. **Table S4.** mean values, SD and coefficient of variation (CV) of biomarker measurements on internal quality control (QC) samples used during the fresh vs frozen measurements with Lumipulse G600-II. Our QC is a pool of 1400 CSF samples belonging to patients affected by neurological and neurodegenerative (mostly AD) diseases (excluding Creutzfeldt-Jacob disease). **Table S5.** Minimum mean relative difference (MMRD) significantly observable with a t-test power above 0.8. The MMRD was calculated considering the measured SD of relative differences and a sample size of 58.

## Data Availability

The raw data used in this study are fully shown in Table S3 in the Supplementary Information.

## Abbreviations

CSF: Cerebrospinal fluid
Aβ40: Amyloid-β 1–40
Aβ42: Amyloid-β 1–42
AD: Alzheimer’s disease
p-tau: Threonine-181-phosphorylated tau
t-tau: Total-tau
MCI: Mild cognitive impairment
NIA-AA: National Institute on Aging and the Alzheimer’s Association
ELISA: Enzyme-linked immunosorbent assay
PET: Positron emission tomography
QC: Quality control
CV: Coefficient of variation
PD: Parkinson’s disease
CI: Confidence interval
ρ: Spearman’s correlation coefficient
α: Linear regression slope
β: Linear regression intercept
