# Supplementary material for: Measurement of CSF core Alzheimer disease biomarkers for routine clinical diagnosis: do fresh vs frozen samples differ?
Source: Alzheimers Res Ther. 2020 Sep 29;12:121. doi: 10.1186/s13195-020-00689-0 (PMC7526419; doi:10.1186/s13195-020-00689-0)
Supplement: Supplementary file 1 — Additional file 1: Table S1. Mean absolute relative differences (MARD) in samples with no cells and 1-2 cells. The measured MARD are higher for samples with 1-2 cells but this difference is significant only for Aβ42. Table S2. Linear regression analysis performed on Mean absolute relative differences (MARD) vs CSF total proteins. No significant correlations were found. Figure S1. Bland-Altman Plot of differences between fresh and frozen CSF aliquots of the same samples vs. the mean of the two measurements (data from Table 1). Shaded areas present 95% confidence interval limits for mean and agreement limits. Table S3. patient sex, patient age, sample internal biobank code, lumbar puncture (LP) date and raw biomarker measurements in fresh and frozen samples. Two aliquots relative to the same CSF sample were measured with Lumipulse G-600 II, one by fresh and the other one after 30 days of storage at -80°C. AD: Alzheimer’s disease; MCI: mild cognitive impairment; MCI-AD: MCI due to AD; p-AD: preclinical AD; CBS: corticobasal syndrome; V-DEM: vascular dementia; PD: Parkinson’s disease; PD-MCI: PD with MCI; PDD: PD with dementia; DLB: dementia with Lewy bodies; FTD: frontotemporal dementia; SMC: subjective memory complains; PSY: psychiatric disease. Table S4. mean values, SD and coefficient of variation (CV) of biomarker measurements on internal quality control (QC) samples used during the fresh vs frozen measurements with Lumipulse G600-II. Our QC is a pool of 1400 CSF samples belonging to patients affected by neurological and neurodegenerative (mostly AD) diseases (excluding Creutzfeldt-Jacob disease). Table S5. Minimum mean relative difference (MMRD) significantly observable with a t-test power above 0.8. The MMRD was calculated considering the measured SD of relative differences and a sample size of 58. [file 13195_2020_689_MOESM1_ESM.docx]

**Supporting Information**

Giovanni Bellomo^1^, Samuela Cataldi^1^, Silvia Paciotti^1,2^, Federico Paolini Paoletti^3^, Davide Chiasserini^2^, Lucilla Parnetti^1^*

^1^Laboratory of Clinical Neurochemistry, Section of Neurology, University of Perugia, Piazzale Lucio Severi 1/8, 06132 Perugia (PG), Italy

^2^Department of Experimental Medicine, Section of Physiology and Biochemistry, University of Perugia, Piazza Lucio Severi 1/8, 06132 Perugia (PG), Italy

^3^Section of Neurology, University of Perugia, Piazzale Lucio Severi 1/8, 06132 Perugia (PG), Italy

*Corresponding Author: Prof. Lucilla Parnetti Section of Neurology, University of Perugia E-mail: lucilla.parnetti@unipg.it

**Contents**

[Table S1 1](#_Toc43906228)

[Table S2 1](#_Toc43906229)

[Figure S1 2](#_Toc43906230)

[Table S3 3](#_Toc43906231)

[Table S4 4](#_Toc43906232)

[Table S5 4](#_Toc43906233)

Table S1 Mean absolute relative differences (MARD) in samples with no cells and 1-2 cells. The measured MARD are higher for samples with 1-2 cells but this difference is significant only for Aβ42.

| Biomarker | MARD (95%CI) 0 cells | MARD (95%CI) 1-2 cells | p-values Wilcoxon test |
| --- | --- | --- | --- |
| Aβ40 | 0.088 (0.068, 0.108) | 0.111 (0.089, 0.135) | 0.12 |
| Aβ42 | 0.064 (0.041, 0.089) | 0.097 (0.068, 0.127) | 0.03 |
| Aβ42/Aβ40 | 0.068 (0.050, 0.086) | 0.065 (0.043, 0.088) | 0.53 |
| t-tau | 0.097 (0.054, 0.141) | 0.122 (0.69, 0.175) | 0.58 |
| p-tau | 0.047 (0.031, 0.062) | 0.043 (0.027, 0.058) | 0.59 |

Table S2 Linear regression analysis performed on Mean absolute relative differences (MARD) vs CSF total proteins. No significant correlations were found.

| MARD vs CSF proteins | Intercept (95%CI) | Slope (95%CI) × 10^-4^ (dL/mg) | Pearson's r |
| --- | --- | --- | --- |
| Aβ40 | 0.08 (0.04, 0.11) | 4.7 (2.5, 11.9) | 0.17 |
| Aβ42 | 0.06 (0.01, 0.11) | 4.6 (-4.5, 14) | 0.13 |
| Aβ42/Aβ40 | 0.07 (0.03, 0.10 | 0.3 (-6.4, 7.0) | 0.01 |
| t-tau | 0.05 (-0.03, 0.14) | 11.6 (-4.1, 27.4) | 0.19 |
| p-tau | 0.05 (0.02, 0.08) | -1.3 (-6.5, 3.9) | -0.07 |


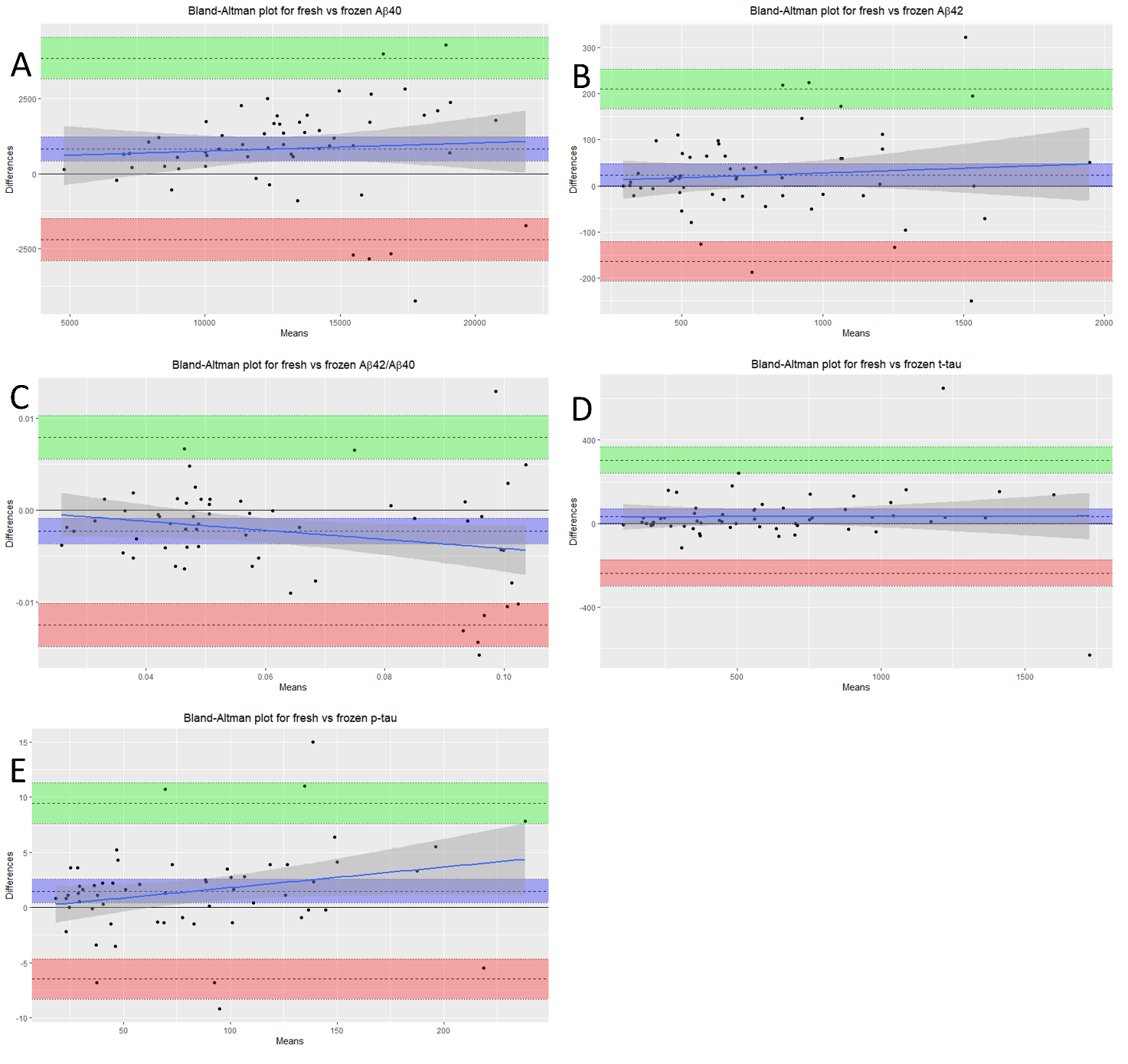
Figure S1 Bland-Altman Plot of differences between fresh and frozen CSF aliquots of the same samples vs. the mean of the two measurements (data from table 1). Shaded areas present 95% confidence interval limits for mean and agreement limits.

Table S3 patient sex, patient age, sample internal biobank code, lumbar puncture (LP) date and raw biomarker measurements in fresh and frozen samples. Two aliquots relative to the same CSF sample were measured with Lumipulse G-600 II, one by fresh and the other one after 30 days of storage at -80°C. AD: Alzheimer’s disease; MCI: mild cognitive impairment; MCI-AD: MCI due to AD; p-AD: preclinical AD; CBS: corticobasal syndrome; V-DEM: vascular dementia; PD: Parkinson’s disease; PD-MCI: PD with MCI; PDD: PD with dementia; DLB: dementia with Lewy bodies; FTD: frontotemporal dementia; SMC: subjective memory complains; PSY: psychiatric disease.

| **Patient data** | | | | | **Fresh** | | | | | **After 1 month freezing** | | | | |
| --- | --- | --- | --- | --- | --- | --- | --- | --- | --- | --- | --- | --- | --- | --- |
| **Sex** | **Age (y)** | **Code** | **Date LP** | **Diagnosis** | **Aβ40 (pg/mL)** | **Aβ42 (pg/mL)** | **Aβ42/Aβ40** | **t-tau (pg/mL)** | **p-tau (pg/mL)** | **Aβ40 (pg/mL)** | **Aβ42 (pg/mL)** | **Aβ42/Aβ40** | **t-tau (pg/mL)** | **p-tau (pg/mL)** |
| F | 74 | 2776 | 16/05/2019 | MCI | 15536 | 773 | 0.05 | 376 | 49.2 | 18203 | 818 | 0.04 | 326 | 44 |
| F | 79 | 2783 | 24/05/2019 | MCI | 14650 | 1540 | 0.11 | 392 | 41.1 | 17480 | 1611 | 0.09 | 320 | 38.9 |
| M | 71 | 2791 | 05/06/2019 | MCI-AD | 14108 | 703 | 0.05 | 590 | 100.2 | 16830 | 726 | 0.04 | 527 | 96.7 |
| M | 73 | 2792 | 06/06/2019 | CBS | 9251 | 783 | 0.08 | 103 | 18.6 | 8703 | 744 | 0.09 | 109 | 17.8 |
| F | 79 | 2795 | 07/06/2019 | MCI | 17467 | 1536 | 0.09 | 344 | 43.4 | 14812 | 1536 | 0.1 | 394 | 44.9 |
| F | 71 | 2799 | 14/06/2019 | MCI-AD | 21632 | 935 | 0.04 | 615 | 90.3 | 19858 | 985 | 0.05 | 677 | 99.5 |
| F | 75 | 2805 | 24/06/2019 | MCI-AD | 20995 | 504 | 0.02 | 673 | 89.2 | 22724 | 631 | 0.03 | 727 | 96 |
| M | 60 | 2810 | 01/07/2019 | V-DEM | 15425 | 1403 | 0.09 | 1410 | 35.3 | 16140 | 1653 | 0.1 | 2040 | 38.7 |
| F | 73 | 2824 | 17/07/2019 | MCI-AD | 15647 | 655 | 0.04 | 761 | 126.5 | 19898 | 842 | 0.04 | 742 | 125.4 |
| F | 80 | 2830 | 25/07/2019 | MCI-AD | 13535 | 689 | 0.05 | 467 | 65.2 | 12974 | 652 | 0.05 | 485 | 66.5 |
| M | 70 | 2834 | 02/08/2019 | PD | 13510 | 1099 | 0.08 | 344 | 44.4 | 12865 | 1040 | 0.08 | 401 | 47.9 |
| M | 61 | 2835 | 07/08/2019 | MCI | 15345 | 1629 | 0.11 | 205 | 24.6 | 14170 | 1434 | 0.1 | 211 | 24.6 |
| M | 72 | 2844 | 29/08/2019 | PD-MCI | 12779 | 1000 | 0.08 | 249 | 34 | 11906 | 854 | 0.07 | 365 | 40.8 |
| F | 74 | 2846 | 09/09/2019 | AD | 21058 | 966 | 0.05 | 1670 | 242.2 | 16759 | 748 | 0.04 | 1534 | 234.4 |
| M | 43 | 2849 | 10/09/2019 | PD | 16330 | 1668 | 0.1 | 366 | 29.7 | 13566 | 1346 | 0.1 | 215 | 29.2 |
| M | 79 | 2852 | 12/09/2019 | AD | 18578 | 1063 | 0.06 | 697 | 89.8 | 14581 | 839 | 0.06 | 623 | 87.5 |
| M | 75 | 2853 | 13/09/2019 | MCI-AD | 18812 | 1151 | 0.06 | 472 | 70 | 15980 | 979 | 0.06 | 429 | 68.7 |
| F | 60 | 2854 | 17/09/2019 | AD | 14744 | 537 | 0.04 | 1169 | 152 | 12797 | 467 | 0.04 | 1006 | 147.9 |
| F | 72 | 2857 | 18/09/2019 | MCI-AD | 14344 | 676 | 0.05 | 593 | 89.7 | 12629 | 585 | 0.05 | 527 | 87.2 |
| F | 69 | 2859 | 19/09/2019 | MCI-AD | 10909 | 540 | 0.05 | 1086 | 132.8 | 9160 | 430 | 0.05 | 984 | 133.7 |
| M | 77 | 2860 | 19/09/2019 | MCI-AD | 15953 | 620 | 0.04 | 1488 | 215.8 | 15018 | 555 | 0.04 | 1336 | 221.3 |
| F | 60 | 2862 | 20/09/2019 | AD | 13587 | 677 | 0.05 | 1541 | 128.8 | 11934 | 580 | 0.05 | 893 | 124.9 |
| M | 79 | 2866 | 26/09/2019 | AD | 13374 | 686 | 0.05 | 972 | 140.1 | 12406 | 621 | 0.05 | 840 | 137.8 |
| M | 58 | 2867 | 27/09/2019 | SMC | 10143 | 991 | 0.1 | 174 | 21.9 | 9895 | 1010 | 0.1 | 167 | 24.1 |
| M | 78 | 2869 | 30/09/2019 | p-AD | 19406 | 1094 | 0.06 | 824 | 90.3 | 18700 | 1035 | 0.06 | 682 | 90.2 |
| F | 70 | 2875 | 07/10/2019 | AD | 13569 | 457 | 0.03 | 705 | 140.2 | 11059 | 359 | 0.03 | 715 | 129.2 |
| F | 75 | 2940 | 06/12/2019 | AD | 7400 | 351 | 0.05 | 572 | 46.1 | 7196 | 356 | 0.05 | 392 | 43.9 |
| F | 78 | 2941 | 09/12/2019 | AD | 11857 | 497 | 0.04 | 1061 | 144.5 | 11293 | 481 | 0.04 | 1025 | 144.7 |
| F | 74 | 2942 | 10/12/2019 | AD | 19652 | 506 | 0.03 | 1376 | 198.9 | 17551 | 484 | 0.03 | 1349 | 193.4 |
| F | 56 | 2943 | 11/12/2019 | MCI | 4875 | 316 | 0.06 | 184 | 24.5 | 4725 | 315 | 0.07 | 184 | 23.4 |
| F | 61 | 2944 | 11/12/2019 | PSY | 11882 | 1133 | 0.1 | 263 | 30.4 | 10902 | 1154 | 0.11 | 274 | 28.5 |
| F | 73 | 2945 | 12/12/2019 | MCI-AD | 10385 | 321 | 0.03 | 874 | 120.6 | 9768 | 313 | 0.03 | 902 | 116.7 |
| M | 74 | 2948 | 13/12/2019 | MCI-AD | 9099 | 395 | 0.04 | 632 | 68.3 | 8944 | 401 | 0.04 | 541 | 69.7 |
| M | 78 | 2949 | 13/12/2019 | DLB | 6622 | 318 | 0.05 | 261 | 35.4 | 6846 | 339 | 0.05 | 233 | 35.5 |
| M | 66 | 2950 | 16/12/2019 | PD | 12204 | 1189 | 0.1 | 311 | 38.4 | 12564 | 1323 | 0.11 | 323 | 37.3 |
| F | 72 | 2951 | 17/12/2019 | AD | 8509 | 471 | 0.06 | 571 | 74.6 | 9041 | 525 | 0.06 | 585 | 70.7 |
| F | 69 | 2953 | 23/12/2019 | PD-MCI | 12978 | 1245 | 0.1 | 627 | 40.6 | 13882 | 1341 | 0.1 | 385 | 40.3 |
| F | 71 | 2956 | 09/01/2020 | AD | 11798 | 494 | 0.04 | 1178 | 108.1 | 11953 | 573 | 0.05 | 1169 | 105.3 |
| F | 77 | 2957 | 09/01/2020 | MCI-AD | 14936 | 700 | 0.05 | 446 | 77.2 | 13492 | 685 | 0.05 | 431 | 78.1 |
| M | 66 | 2958 | 15/01/2020 | AD | 8452 | 505 | 0.06 | 496 | 82.2 | 7385 | 508 | 0.07 | 497 | 83.7 |
| M | 53 | 2960 | 17/01/2020 | DLB | 14381 | 486 | 0.03 | 624 | 111 | 13003 | 500 | 0.04 | 648 | 110.6 |
| M | 76 | 2963 | 20/01/2020 | MCI-AD | 11274 | 464 | 0.04 | 570 | 100.2 | 10010 | 453 | 0.05 | 550 | 101.6 |
| M | 62 | 2964 | 21/01/2020 | PD-MCI | 13382 | 864 | 0.06 | 212 | 37.3 | 11709 | 846 | 0.07 | 205 | 35.3 |
| M | 76 | 2965 | 22/01/2020 | MCI | 13628 | 1205 | 0.09 | 195 | 29.5 | 11696 | 1202 | 0.1 | 205 | 28.2 |
| F | 75 | 2970 | 23/01/2020 | FTD | 7321 | 634 | 0.09 | 246 | 30.3 | 6662 | 664 | 0.1 | 221 | 26.7 |
| M | 74 | 2974 | 28/01/2020 | AD | 10942 | 293 | 0.03 | 962 | 152.2 | 10110 | 294 | 0.03 | 1003 | 145.8 |
| M | 57 | 2976 | 30/01/2020 | SMC | 12854 | 1251 | 0.1 | 339 | 27 | 11521 | 1171 | 0.1 | 181 | 23.4 |
| F | 79 | 2977 | 31/01/2020 | MCI-AD | 12466 | 559 | 0.04 | 451 | 74.8 | 10197 | 498 | 0.05 | 442 | 64.1 |
| F | 67 | 2978 | 31/01/2020 | PD | 13578 | 1267 | 0.09 | 208 | 31.6 | 12228 | 1155 | 0.09 | 205 | 30 |
| F | 77 | 2979 | 04/02/2020 | AD | 14644 | 738 | 0.05 | 985 | 136.6 | 13799 | 701 | 0.05 | 954 | 136.8 |
| M | 70 | 2980 | 04/02/2020 | PD-MCI | 8634 | 811 | 0.09 | 188 | 23.5 | 8384 | 780 | 0.09 | 160 | 22.7 |
| M | 60 | 2982 | 05/02/2020 | CBS | 10378 | 473 | 0.05 | 702 | 102.4 | 9651 | 460 | 0.05 | 703 | 100.8 |
| F | 69 | 2984 | 06/02/2020 | MCI | 15074 | 848 | 0.06 | 374 | 51.7 | 14147 | 869 | 0.06 | 372 | 50.1 |
| M | 65 | 2986 | 07/02/2020 | AD | 8901 | 487 | 0.05 | 773 | 101.6 | 7694 | 468 | 0.06 | 747 | 98.9 |
| M | 72 | 2991 | 13/02/2020 | MCI-AD | 19083 | 704 | 0.04 | 1239 | 189.1 | 17130 | 685 | 0.04 | 1208 | 185.8 |
| F | 77 | 2999 | 20/02/2020 | SMC | 20252 | 1972 | 0.1 | 366 | 49.6 | 17875 | 1922 | 0.11 | 354 | 45.3 |
| M | 76 | 3000 | 20/02/2020 | PDD | 7539 | 359 | 0.05 | 336 | 58.5 | 6871 | 332 | 0.05 | 360 | 56.4 |
| F | 69 | 3001 | 21/02/2020 | MCI-AD | 16938 | 598 | 0.04 | 909 | 146.2 | 15226 | 616 | 0.04 | 843 | 131.2 |

Table S4 mean values, SD and coefficient of variation (CV) of biomarker measurements on internal quality control (QC) samples used during the fresh vs frozen measurements with Lumipulse G600-II. Our QC is a pool of 1400 CSF samples belonging to patients affected by neurological and neurodegenerative (mostly AD) diseases (excluding Creutzfeldt-Jacob disease).

|  | **QC** | | |
| --- | --- | --- | --- |
| **Biomarker** | **Mean** | **SD** | **CV** |
| Aβ40 | 7887 | 488 | 0.08 |
| Aβ42 | 488 | 32 | 0.07 |
| Aβ42/Aβ40 | 0.0621 | 0.0035 | 0.06 |
| p-tau | 52.7 | 2.9 | 0.06 |
| t-tau | 407 | 34 | 0.08 |

Table S5 Minimum mean relative difference (MMRD) significantly observable with a t-test power above 0.8. The MMRD was calculated considering the measured SD of relative differences and a sample size of 58.

| Biomarker | MMRD |
| --- | --- |
| Aβ40 | 4.0% |
| Aβ42 | 4.5% |
| Ab42/Ab40 | 3.0% |
| p-tau | 2.5% |
| t-tau | 6.0% |
